# Real-life effects of dupilumab in patients with severe type 2 asthma, according to atopic trait and presence of chronic rhinosinusitis with nasal polyps

**DOI:** 10.3389/fimmu.2023.1121237

**Published:** 2023-03-30

**Authors:** Corrado Pelaia, Alida Benfante, Maria Teresa Busceti, Maria Filomena Caiaffa, Raffaele Campisi, Giovanna Elisiana Carpagnano, Nunzio Crimi, Maria D’Amato, Maria Pia Foschino Barbaro, Angelantonio Maglio, Elena Minenna, Santi Nolasco, Giuseppe Paglino, Francesco Papia, Girolamo Pelaia, Andrea Portacci, Luisa Ricciardi, Nicola Scichilone, Giulia Scioscia, Massimo Triggiani, Giuseppe Valenti, Alessandro Vatrella, Claudia Crimi

**Affiliations:** ^1^ Department of Health Sciences, University “Magna Graecia” of Catanzaro, Catanzaro, Italy; ^2^ Dipartimento di Promozione della Salute, Materno Infantile, Medicina Interna e Specialistica di Eccellenza (PROMISE), University of Palermo, Palermo, Italy; ^3^ Department of Medical and Surgical Sciences, University of Foggia, Foggia, Italy; ^4^ Department of Clinical and Experimental Medicine, University of Catania, Catania, Italy; ^5^ Department of Basic Medical Science, Neuroscience and Sense Organs, University “Aldo Moro”, Bari, Italy; ^6^ Department of Respiratory Medicine, University “Federico II” of Naples, Naples, Italy; ^7^ Department of Medicine, Surgery and Dentistry, University of Salerno, Salerno, Italy; ^8^ Allergology and Pulmonology Unit, Provincial Outpatient Center of Palermo, Palermo, Italy; ^9^ Department of Clinical and Experimental Medicine, University of Messina, Messina, Italy

**Keywords:** severe asthma, nasal polyps, interleukin 4, interleukin 13, dupilumab, clinical remission

## Abstract

**Background:**

The efficacy of dupilumab as biological treatment of severe asthma and chronic rhinosinusitis with nasal polyps (CRSwNP) depends on its ability to inhibit the pathophysiologic mechanisms involved in type 2 inflammation.

**Objective:**

To assess in a large sample of subjects with severe asthma, the therapeutic impact of dupilumab in real-life, with regard to positive or negative skin prick test (SPT) and CRSwNP presence or absence.

**Methods:**

Clinical, functional, and laboratory parameters were measured at baseline and 24 weeks after the first dupilumab administration. Moreover, a comparative evaluation was carried out in relation to the presence or absence of SPT positivity and CRSwNP.

**Results:**

Among the 127 recruited patients with severe asthma, 90 had positive SPT, while 78 reported CRSwNP. Compared with the 6 months preceding the first dupilumab injection, asthma exacerbations decreased from 4.0 (2.0-5.0) to 0.0 (0.0-0.0) (p < 0.0001), as well as the daily prednisone intake fell from 12.50 mg (0.00-25.00) to 0.00 mg (0.00-0.00) (p < 0.0001). In the same period, asthma control test (ACT) score increased from 14 (10-18) to 22 (20-24) (p < 0.0001), and sino-nasal outcome test (SNOT-22) score dropped from 55.84 ± 20.32 to 19.76 ± 12.76 (p < 0.0001). Moreover, we observed relevant increases in forced expiratory volume in one second (FEV1) from the baseline value of 2.13 L (1.62-2.81) to 2.39 L (1.89-3.06) (p < 0.0001). Fractional exhaled nitric oxide (FeNO) values decreased from 27.0 ppb (18.0-37.5) to 13.0 ppb (5.0-20.0) (p < 0.0001). These improvements were quite similar in subgroups of patients characterized by SPT negativity or positivity, and CRSwNP absence or presence. No statistically significant correlations were detected between serum IgE levels, baseline blood eosinophils or FeNO levels and dupilumab-induced changes, with the exception of FEV1 increase, which was shown to be positively correlated with FeNO values (r = 0.3147; p < 0.01).

**Conclusion:**

Our results consolidate the strategic position of dupilumab in its role as an excellent therapeutic option currently available within the context of modern biological treatments of severe asthma and CRSwNP, frequently driven by type 2 airway inflammation.

## Introduction

Dupilumab is a completely human monoclonal antibody, belonging to the IgG4 immunoglobulin class, whose mechanism of action consists of the dual antagonism of the interleukin 4 (IL-4) and 13 (IL-13) receptors (1). In addition to the treatment of severe asthma and atopic dermatitis, dupilumab is also indicated for the biological therapy of nasal polyposis, which is a frequent comorbidity of severe asthma (2).

IL-4 and IL-13 play critical roles in the pathogenesis of severe type 2 asthma (3). In particular, IL-4 is crucial in the development and maintenance of the acquired immune response mediated by T helper 2 (Th2) lymphocytes. At the level of B lymphocytes of allergic patients, IL-4 and IL-13 induce the so-called isotypic switch, responsible for the synthesis of immunoglobulins E (IgE), which degranulate mast cells and basophils, facilitate the presentation of allergens by dendritic cells to T lymphocytes, and inhibit eosinophil apoptosis (4). IL-4 and IL-13 promote the trafficking of eosinophils to inflammatory sites and impair the integrity of the airway epithelial barrier. Furthermore, IL-13 stimulates mucus secretion and goblet cell hyperplasia, and also up-regulates the expression of the inducible form of the enzyme nitric oxide (NO) synthase (iNOS), which increases NO production within the airways (5, 6). Chronic rhinosinusitis with nasal polyps (CRSwNP) is a frequent comorbidity of severe asthma, and type 2 inflammation very often contributes significantly to the pathogenesis of nasal polyposis. Indeed, at the level of upper airways IL-4 and IL-13 play a key role in both inflammatory and structural changes (tissue remodelling) that underlie the formation of nasal polyps (7).

The efficacy of dupilumab as biological treatment of severe asthma and nasal polyposis depends on its remarkable ability to inhibit the pathophysiologic mechanisms involved in type 2 inflammation. In fact, dupilumab is an efficient dual antagonist of both IL-4 and IL-13 receptors 13 (3). Specifically, dupilumab binds with high affinity to the IL-4 receptor α subunit (IL-4Rα). This receptor subunit is a key component of the type I receptor, consisting of the IL-4Rα/γC dimer, which is activated by IL-4 (8). The type II receptor is instead constituted by the IL-4Rα subunit and the α1 chain of the IL-13 receptor (IL-4Rα/IL-13Rα1 dimer), and can therefore be stimulated by IL-4 and IL-13. The type I receptor is predominantly expressed by immune-inflammatory cells such as T and B lymphocytes, dendritic cells, monocytes/macrophages, mast cells, basophils and eosinophils. The type II receptor is also present on airway structural cells such as goblet cells, fibroblasts and smooth muscle cells (9). Upon pharmacological blockade of both type I and type II receptors, dupilumab neutralizes the biological effects of IL-4 and IL-13.

Due to this powerful mechanism of action, within the context of add-on biological treatment of severe asthma and nasal polyposis dupilumab exerts remarkable therapeutic effects, well documented by several randomized controlled trials (RCTs) (10). In particular, the “LIBERTY ASTHMA QUEST” study demonstrated that dupilumab was capable of significantly reducing the annual rate of severe asthma exacerbations and improving lung function (11). Furthermore, the “LIBERTY ASTHMA VENTURE” trial highlighted the ability of dupilumab to significantly decrease the consumption of oral corticosteroids (OCS) (12). These results were recently confirmed by the open-label extension study LIBERTY ASTHMA TRAVERSE, which further monitored for additional 96 weeks many patients previously enrolled in the LIBERTY ASTHMA QUEST and LIBERTY ASTHMA VENTURE (13). As regards the adjunctive biological therapy of nasal polyposis, the studies “LIBERTY NP SINUS-24” and “LIBERTY NP SINUS-52” documented the efficacy of dupilumab by evaluating the improvement of many relevant parameters (14).

However, only a few real-world studies referring to a quite low number of patients have been published so far (15–19). Hence, the aim of our present real-life observational investigation was to evaluate, in a larger sample of subjects with severe asthma, also including many patients with nasal polyposis, the therapeutic impact of dupilumab on upper and lower airway symptoms, severe asthma exacerbations, OCS intake and lung function, as well as on the overall clinical expression of nasal polyposis.

## Patients and methods

### Study design and patient enrollment

In the present retrospective multicenter observational study, we recruited adult outpatients (>18 years) with severe type 2 asthma treated with dupilumab. Subjects were enrolled at the following asthma centers: Respiratory Medicine Section, University “Aldo Moro”, Bari, Italy; Allergy and Respiratory Medicine, University of Catania, Italy; Respiratory Disease Unit, University “Magna Graecia” of Catanzaro, Italy; Allergology and Clinical Immunology Unit, University of Foggia, Italy; Respiratory Disease Unit, University of Foggia, Italy; Allergy and Clinical Immunology Unit, University of Messina; Pulmonology Unit, “Monaldi” University Hospital, Naples, Italy; Pulmonology Unit, University of Palermo, Italy; Allergology and Pulmonology Unit, Provincial Outpatient Center of Palermo, Italy; Respiratory Disease Unit, University of Salerno, Italy; Division of Allergy and Clinical Immunology, University of Salerno.

Patients reported persistent asthmatic symptoms and required high doses of the inhaled therapeutic combinations ICS (inhaled corticosteroids)/LABA (long-acting β_2_-adrenergic agonists), associated with a LAMA (long-acting muscarinic receptor antagonist). Enrollment took place consecutively, and the only inclusion criteria were those needed for prescription of dupilumab. All recruited patients met the European Respiratory Society (ERS)/American Thoracic Society (ATS) criteria defining severe uncontrolled asthma (20). Blood counts of eosinophils, basophils and neutrophils were obtained using automated hematology analyzers (21, 22). At baseline, all participants had an eosinophilic blood count of at least 150 cells/μL and/or fractional exhaled nitric oxide (FeNO) levels greater than 25 parts per billion (ppb), and/or they were treated with lifelong or near-continuous OCS therapies.

The aforementioned centers participating in the study used a shared database to acquire clinical, functional and biological data. Smoking habit and comorbidities such as gastroesophageal reflux disease (GERD), nasal polyposis, bronchiectasis, osteoporosis, anxiety, atopic dermatitis and obstructive sleep apnea syndrome (OSAS) were evaluated. Symptom control was assessed by administering to all recruited patients the asthma control test (ACT). The latter includes 5 key questions referring to the frequency of asthma symptoms and to the need of inhaled rescue medication during the previous 4 weeks (23). Each question scores from 1 to 5; therefore, ACT score ranges from 5 (worse control) to 25 points (complete control). Spirometry was performed following ATS/ERS guidelines (24). FeNO levels were measured in accordance with ATS/ERS recommendations (25, 26). Treatment with dupilumab was prescribed according to current eligibility guidelines, and the drug was administered subcutaneously using an initial dose of 600 mg (two 300 mg injections at different skin sites), followed by a maintenance dose of 300 mg every 2 weeks (27, 28).

This observational study met the standards of Good Clinical Practice (GCP) and the principles of the Declaration of Helsinki. All recruited patients signed a written informed consent. Our study was also conducted in accordance with the provisions of the local Ethics Committee of Calabria Region, Italy (Catanzaro, Italy; document n. 182 – 20 May 2021).

### Outcomes and measurements

The main purpose of this real-life study was to evaluate the efficacy of dupilumab in daily clinical practice. The number of asthma exacerbations, emergency department visits and daily inhalations of short-acting β_2_-adrenergic agonists (SABA), as well as prednisone intake, ACT score, sino-nasal outcome test questionnaire (SNOT-22), the number of relapses of nasal polyposis, forced expiratory volume in one second (FEV_1_), forced vital capacity (FVC), mean forced expiratory flow between 25% and 75% of FVC (FEF_25-75_), FeNO levels, as well as blood eosinophil, basophil, and neutrophil counts were assessed at baseline and 24 weeks after the first dupilumab administration.

A secondary objective was to retrospectively verify the therapeutic responses of our patients to dupilumab, in relation to SPT positivity or negativity, as well as with regard to the presence or absence of CRSwNP. The diagnosis of CRSwNP was formulated on the basis of symptoms, nasal endoscopy and computed tomography (CT) (29, 30). Skin prick test (SPT) was performed by placing a drop of each allergen on the forearm evidenced with a skin marker, and each drop was pricked by a sterile lancet; skin sensitivity was determined by comparing any wheal with that one caused by histamine (31).

Furthermore, after 6 months of adjunctive therapy with dupilumab we analyzed the possible correlations existing between the baseline concentrations of serum IgE, FeNO and blood eosinophils, and the observed changes regarding asthma exacerbations, daily consumption of prednisone and SABA, ACT score, SNOT-22 score, FEV_1_, FVC, and FEF_25-75_ values.

In addition, the occurrence of unwanted side effects was investigated on the basis of available information stored in clinical records.

### Statistical analysis

All data are expressed as mean ± standard deviation (SD) if normally distributed, otherwise as median values ​​with the interquartile range (IQR). The normality of data distribution was checked using Anderson-Darling and Kolmogorov-Smirnov tests. Paired t-test and Mann-Whitney’s U-test for paired data were used to compare variables when appropriate. The latter statistical test was also used for the secondary objective of the study, i.e. the comparative evaluation of dupilumab efficacy in patients with positive or negative SPT, and with regard to the presence or absence of CRSwNP. Fisher’s test was applied to compare categorical variables. The association between baseline concentrations of type 2 inflammation biomarkers (serum IgE, blood eosinophils, and FeNO) and changes in clinical and functional parameters was assessed using linear regression analysis. In particular, the correlation index R for Spearman’s ranks was evaluated. A p-value less than 0.05 (two-tailed) was considered as statistically significant. Statistical analyses and figures were performed using Prism Version 9.4.0 software (GraphPad Software Inc., San Diego, California, USA).

## Results

### Efficacy of dupilumab in the whole population

A total of 127 participants were recruited, including 63 (49.6%) women and 64 (50.4%) men, with a median age of 56.0 years (45.0-64.0), and a median body mass index (BMI) value of 26.0 Kg/m^2^ (23.0-30.0). Mean baseline FEV_1_ was 76.56 ± 20.03% of predicted value. Among the enrolled patients, 90 (70.9%) had positive SPT for perennial and/or seasonal allergens, while 78 (61.4%) reported CRSwNP. Baseline patient characteristics are summarized in **Table 1**.

**Table 1:** Baseline patient characteristics, stratified according SPT negativity or positivity and CRSwNP absence or presence.

Characteristic	Overall N = 127	SPT - N = 37	SPT + N = 90	p	CRSwNP - N = 49	CRSwNP + N = 78	p
**Female gender, N (%)**	63 (49.6)	13 (35.1)	50 (55.6)	< 0.05	27 (55.1)	36 (46.2)	0.3651
**Male gender, N (%)**	64 (50.4)	24 (64.9)	40 (44.4)	< 0.05	22 (44.9)	42 (53.8)	0.3651
**Age, median values (IQR), years**	56.0 (45.0-64.0)	59.0 (54.0-69.0)	55.0 (43.0-63.3)	< 0.05	54.0 (45.0-63.0)	56.5 (45.0-65.3)	0.6326
**Age of asthma onset, median values (IQR), years**	30.0 (20.0-44.0)	44.0 (31.5-58.0)	25.5 (18.0-40.0)	< 0.0001	35.0 (20.0-48.5)	30.0 (19.8-42.0)	0.5924
**Duration of asthma, median values (IQR), years**	17.0 (10.0-30.0)	10.0 (8.0-20.0)	20.0 (14.0-33.0)	< 0.01	10.0 (6.0-21.0)	20.0 (14.0-33.3)	< 0.001
**BMI, median values (IQR), Kg/m^2^**	26.0 (23.0-30.0)	25.6 (23.3-27.0)	27.0 (23.2-30.1)	0.2436	27.0 (25.0-31.1)	25.2 (23.0-28.4)	0.1730
**Exacerbations, median values (IQR), N**	4.0 (2.0-5.0)	3.5 (2.0-5.0)	4.0 (2.0-5.0)	0.6830	3.0 (2.0-5.0)	4.0 (2.0-5.0)	0.1124
**Prednisone, median values (IQR), mg/day**	12.50 (0.00-25.00)	6.75 (0.00-12.50)	12.50 (0.00-25.00)	0.0899	5.00 (0.00-12.50)	12.50 (3.75-25.00)	< 0.01
**ACT score, median values (IQR)**	14 (10-18)	16 (14-18)	13 (9-17)	< 0.01	15 (11-18)	14 (10-17)	0.2057
**FEV_1_, mean values (SD), % predicted**	76.56 (20.03)	77.75 (17.64)	76.07 (21.01)	0.6730	76.28 (17.41)	76.73 (21.58)	0.9038
**FeNO, median values, (IQR), ppb**	27.0 (18.0-37.5)	20.0 (9.5-33.0)	29.0 (20.0-40.0)	0.0675	29.0 (19.0-44.0)	25.0 (16.0-35.0)	0.3369
**Blood eosinophils, median values, (IQR), cells/μL**	400.0 (210.0-680.0)	410.0 (177.5-780.0)	399.0 (230.0-610.0)	0.9638	415.0 (222.3-734.8)	399.0 (200.0-640.0)	0.6567
**Gastro-esophageal reflux disease, N (%)**	56 (44.1)	15 (40.5)	41 (45.6)	0.6954	19 (38.8)	37 (47.4)	0.3640
**Atopy, N (%)**	90 (70.9)	0 (0.00)	90 (100)	< 0.0001	35 (71.4)	55 (70.5)	> 0.9999
**CRSwNP, N (%)**	78 (61.4)	23 (62.2)	55 (61.1)	> 0.9999	0 (0.00)	78 (100)	< 0.0001
**Bronchiectasis, N (%)**	22 (17.3)	7 (18.9)	15 (16.7)	0.7987	4 (8.2)	18 (23.1)	< 0.05
**Osteoporosis, N (%)**	25 (19.7)	7 (18.9)	18 (20.0)	0.6410	5 (10.2)	20 (25.6)	< 0.05
**Anxiety, N (%)**	32 (25.2)	7 (18.9)	25 (27.8)	0.3713	5 (10.2)	27 (34.6)	< 0.01
**Dermatitis, N (%)**	14 (11.0)	0 (0.00)	14 (15.6)	< 0.05	6 (12.2)	8 (10.3)	0.7755
**Obstructive sleep apnea syndrome, N (%)**	13 (10.2)	0 (0.00)	13 (14.4)	< 0.05	6 (12.2)	7 (8.9)	0.5618

SPT, skin prick test; CRSwNP, chronic rhinosinusitis with nasal polyps; IQR, interquartile range; SD, standard deviation; BMI, body mass index; ACT, asthma control test; FEV1, forced expiratory volume in one second; FeNO, fractional exhaled nitric oxide.

**Figure 1:**
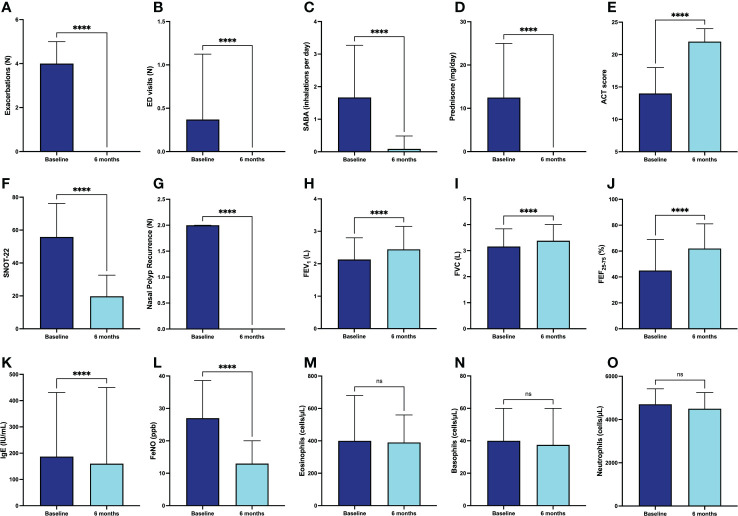
Efficacy of dupilumab in the whole population of patients with severe asthma, with regard to asthma exacerbations **(A)**, ED visits **(B)**, daily SABA inhalations **(C)**, prednisone intake **(D)**, ACT score **(E)**, SNOT-22 **(F)**, nasal polyp recurrence **(G)**, FEV1 **(H)**, FVC **(I)**, FEF25-75 **(J)**, IgE **(K)**, FeNO **(L)**, blood eosinophils **(M)**, blood basophils **(N)**, and blood neutrophils **(O)**. Values of ED visits, daily SABA inhalations and SNOT-22 are expressed as mean (± SD). All other parameters are expressed as median values (IQR). **** p < 0.0001; ns, not significant.

Compared with the 6-month pre-treatment period (before the first injection of dupilumab), the median number of asthma exacerbations dramatically decreased from 4.0 (2.0-5.0) to 0.0 (0.0-0.0) (p < 0.0001) after 6 months of anti-IL4R/IL-13R therapy (**Figure 1A**). Furthermore, in the same period the mean number of emergency department (ED) visits (0.40 ± 0.75 vs. 0.0 ± 0.0; p < 0.0001) (**Figure 1B**) and daily SABA inhalations (1.67 ± 1.59 vs. 0.09 ± 0.39; p < 0.0001) also significantly fell down (**Figure 1C**). These therapeutic effects allowed a reduction of the daily prednisone intake from 12.50 mg (0.00-25.00) to 0.00 mg (0.00-0.00) (p < 0.0001) (**Figure 1D**). In addition, the percentage of patients taking daily OCS decreased from 66.9% (before starting dupilumab treatment) to 5.5% after six months of therapy. After 24 weeks of treatment with dupilumab, ACT score increased significantly from a baseline value of 14 (10-18) to 22 (20-24) (p < 0.0001) (**Figure 1E**), and SNOT-22 score dropped from 55.84 ± 20.32 to 19.76 ± 12.76 (p < 0.0001) (**Figure 1F**) in subjects also suffering from nasal polyposis. Furthermore, in this subset of patients the number of recurrences of nasal polyposis decreased from 2 (1-2.5) to 0 (0-0) (p < 0.0001) after initiation of dupilumab therapy (**Figure 1G**). In addition to the above clinical results, we also observed a significant improvement in respiratory function, documented by increases in FEV_1_ from the baseline value of 2.13 L (1.62-2.81) to 2.39 L (1.89-3.06) (p < 0.0001) (**Figure 1H**), in FVC from 3.16 L (2.43-3.84) to 3.36 L (2.68-3.92) (p < 0.0001) (**Figure 1I**), and in FEF_25-75_ from 46.50% (30.75-69.00) to 63.50% (44.75-81.25) (p < 0.0001) of predicted values ​​(**Figure 1J**). Furthermore, serum IgE levels lowered from 238.0 IU/mL (81.0-499.0) to 160.0 IU/mL (49.0-450.0) (p < 0.0001) (**Figure 1K**). In the same observation period FeNO values ​​decreased from 27.0 ppb (18.0-37.5) to 13.0 ppb (5.0-20.0) (p < 0.0001) (**Figure 1L**). Regarding the possible hematological effects of dupilumab, after six months of additional treatment, the blood eosinophil count did not undergo substantial variations, thus changing from 400.0 cells/µL (222.5-677.5) to 395.0 cells/µL (181.5-565.0) (p = 0.427) (**Figure 1M**). Similarly, blood basophil and neutrophil values ​​did not change significantly, going from 40.0 cells/µL (30.0-60.0) to 37.5 cells/µL (20.0-60.0) (p = 0.068) (**Figure 1N**), and from 4705.0 cells/µL (3635.0-5423.0) to 4500.0 cells/µL (4000.0-5250.0) (p = 0.396) (**Figure 1O**), respectively.

Moreover, after a six-month treatment with dupilumab, when considering the key variables of clinical remission that include evaluation of asthma symptoms (ACT score ≥20), optimization of lung function (FEV_1_ ≥80% of predicted value), zeroing of exacerbations and OCS (zero exacerbations and zero OCS use) (32, 33), 47.24% of enrolled patients satisfied these criteria.

### Efficacy of dupilumab in different type 2 asthma phenotypes

**Figure 2:**
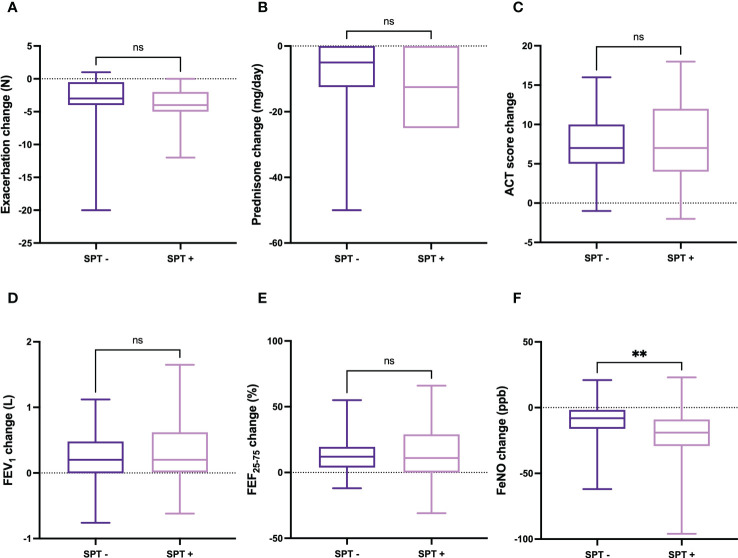
Comparative evaluation of dupilumab effects in relation to SPT negativity or positivity, with regard to asthma exacerbations **(A)**, prednisone intake **(B)**, ACT score **(C)**, FEV1 **(D)**, FEF25-75 **(E)**, and FeNO levels **(F)**. Boxes display median values and IQR, and whiskers define maximum and minimum. ns, not significant; ** p < 0.01.

Improvements in clinical, functional, and hematological parameters after six months of dupilumab treatment were quite similar in subgroups of patients characterized by SPT negativity or positivity, respectively. Specifically, the decrease in the number of asthma exacerbations was -3.00 (from -4.00 to -0.50) in patients with negative SPT and -4.00 (from -5.00 to -2.00) in subjects with positive SPT, respectively (p = 0.158) (**Figure 2A**). The daily dose of prednisone decreased by -5.00 mg (from -12.50 to 0.00) in patients with negative SPT and -12.5 mg (from -25.00 to 0.00) in subjects with positive SPT, respectively (p = 0.136) (**Figure 2B**). The median changes in ACT score were 7 points (5-10) and 7 points (4-12) in patients with negative and positive SPT, respectively (p = 0.690) (**Figure 2C**). The mean increase in FEV_1_ was 0.20 L (0.00-0.48) in patients with negative SPT and 0.20 L (0.01-0.62) in subjects with positive SPT; this difference was not statistically significant (p = 0.409) (**Figure 2D**). Six months after the first dupilumab injection, the increase in FEF_25-75_ was 12.00% (3.75-19.50) in patients with negative SPT and 11.00% (0.00-29.00) in subjects with positive SPT, respectively (p = 0.827) (**Figure 2E**). Furthermore, the reduction of FeNO levels ​​was -8.00 ppb (from -16.17 to -1.75) in patients with negative SPT and -19.00 ppb (from -29.25 to -9.00) in subjects with positive SPT, but in this case the difference overcame the threshold of statistical significance (p < 0.01) (**Figure 2F**).

**Figure 3:**
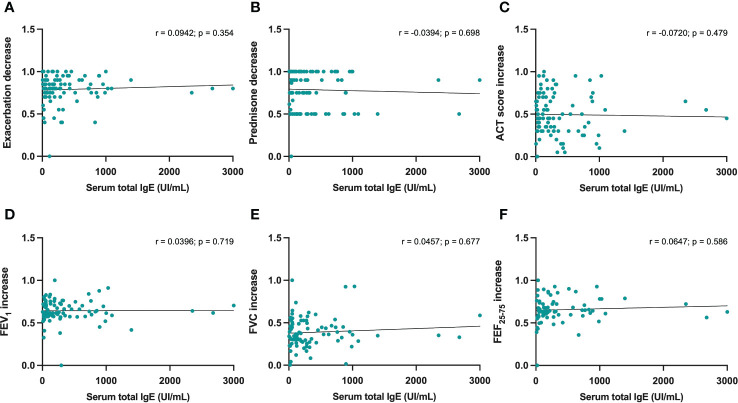
Correlations between serum IgE concentrations and 6-month changes induced by dupilumab, with regard to asthma exacerbations **(A)**, prednisone intake **(B)**, ACT score **(C)**, FEV1 **(D)**, FVC **(E)**, and FEF25-75 **(F)**.

**Figure 4:**
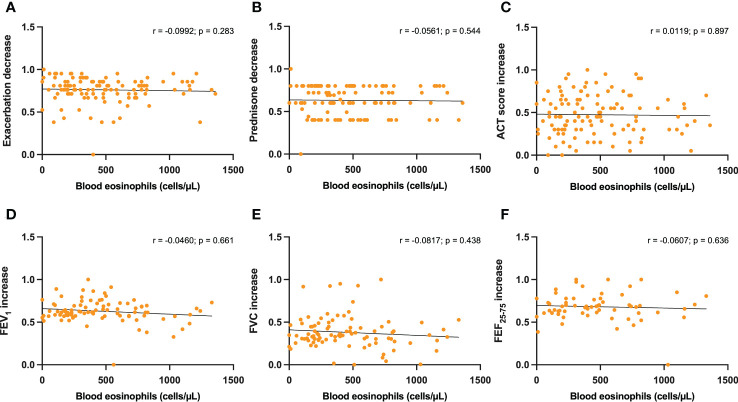
Correlations between blood eosinophils and 6-month changes induced by dupilumab, with regard to asthma exacerbations **(A)**, prednisone intake **(B)**, ACT score **(C)**, FEV1 **(D)**, FVC **(E)**, and FEF25-75 **(F)**.

No statistically significant correlations were detected between either serum IgE levels or baseline blood eosinophils, and dupilumab-induced changes in the following parameters: reduction in asthma exacerbations (**Figures 3A**, **4A**), decrease in daily prednisone dose (**Figures 3B**, **4B**), increases in ACT score (**Figures 3C**, **4C**), FEV_1_ (**Figures 3D**, **4D**), FVC (**Figures 3E**, **4E**), and FEF_25-75_ (**Figures 3F**, **4F**).

**Figure 5:**
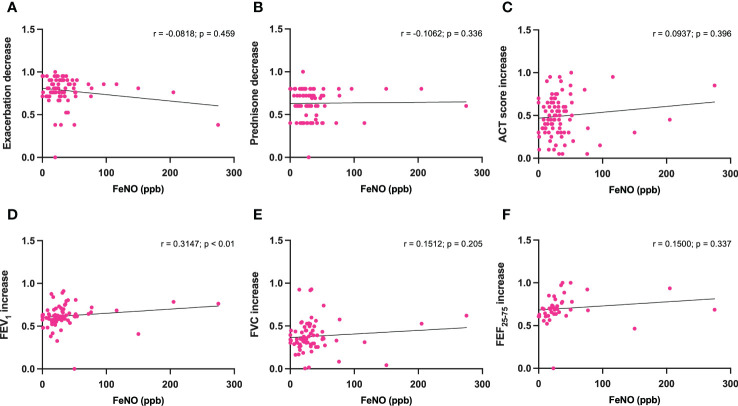
Correlations between FeNO levels and 6-month changes induced by dupilumab, with regard to asthma exacerbations **(A)**, prednisone intake **(B)**, ACT score **(C)**, FEV1 **(D)**, FVC **(E)**, and FEF25-75 **(F)**.

In addition, when considering the above parameters, no correlations were also found between baseline FeNO levels and dupilumab-induced changes (**Figures 5A–C, E, F**), with the exception of FEV_1_ increases, which were shown to be positively correlated with FeNO values (r = 0.3147; p < 0.01) (**Figure 5D**).

### Efficacy of dupilumab in patients with or without CRSwNP

**Figure 6:**
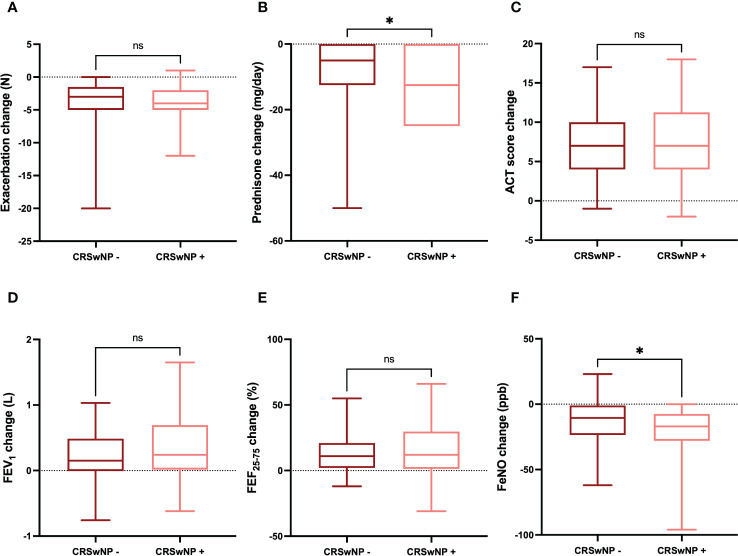
Comparative evaluation of dupilumab effects in relation to CRSwNP absence or presence, with regard to asthma exacerbations **(A)**, prednisone intake **(B)**, ACT score **(C)**, FEV1 **(D)**, FEF25-75 **(E)**, and FeNO levels **(F)**. Boxes display median values and IQR, and whiskers define maximum and minimum. ns, not significant; * p < 0.05.

The improvements in clinical and functional parameters observed after six months of treatment with dupilumab were quite similar in the subgroups of patients characterized by the absence or presence of CRSwNP, respectively. In particular, the decrease in the number of asthma exacerbations was -3.00 (-5.00 to -1.50) in patients without CRSwNP and -4.00 (-5.00 to -2.00) in subjects with CRSwNP, respectively (p = 0.413) (**Figure 6A**). The daily dose of prednisone decreased by -5.00 mg (from -12.50 to 0.00) in patients without CRSwNP and -12.5 mg (from -25.00 to 0.00) in subjects with CRSwNP, respectively (p < 0.05) (**Figure 6B**). The increase in ACT score was 7 points (4-10) and 7 points (4-11) in patients without or with CRSwNP, respectively (p = 0.422) (**Figure 6C**). The mean increase in FEV_1_ was 0.15 L (-0.01-0.49) in patients without CRSwNP, and 0.24 L (0.01-0.69) in subjects with CRSwNP; this difference was not statistically significant (p = 0.138) (**Figure 6D**). Six months after the first dupilumab injection, the increase in FEF_25-75_ was 11.00% (2.00-21.00) in patients without CRSwNP and 12.00% (1.25-29.75) in subjects with CRSwNP, respectively (p = 0.479) (**Figure 6E**). Furthermore, the reduction in FeNO levels was -10.51 ppb (-23.50 to -1.00) in patients without CRSwNP and -17.00 ppb (-28.00 to -7.50) in subjects with CRSwNP, respectively, but in this case the difference reached the threshold of statistical significance (p < 0.05) (**Figure 6F**).

### Safety and tolerability profile of dupilumab

Add-on biological treatment with dupilumab was well tolerated, and no serious adverse reactions were observed during this real-world investigation. With regard to mild and transient side effects, increases in blood eosinophil counts with no symptoms were found in 5 (3.94%) subjects, 4 (3.15%) cases of conjunctivitis were reported, 2 (1.57%) injection site reactions were detected, and 1 (0.79%) patient experienced headache. All these mild side effects remitted spontaneously and did not require any specific treatment.

## Discussion

Taken together, the results of the present multicenter real-life study, performed in patients with severe asthma and frequent nasal polyposis, show that dupilumab induced relevant therapeutic effects. Firstly, six months of treatment with this biologic drug cleared asthma exacerbations. This made it possible to effectively prevent any access to the emergency room, the use of short-acting bronchodilators as needed, and the intake of OCS. The latter aspect is of considerable importance as it further exceeds the efficacy data reported by the Liberty Asthma VENTURE trial (12). Indeed, VENTURE authors reported that 52.4% of patients interrupted OCS after 24 weeks of treatment with dupilumab, whereas after the same period of time OCS withdrawal was achieved by 92.9% of our steroid-dependent patients. The relevance of zeroing the use of OCS is closely related to the possibility of abrogating the well-known side effects of oral corticosteroid therapy, including adrenal insufficiency, respiratory infections, diabetes mellitus, arterial hypertension, osteoporosis, glaucoma and cataract (34, 35).

When compared to QUEST (Q) and VENTURE (V) trials, other important differences with our real-life study regard the baseline characteristics of recruited patients. In particular, we enrolled a population of asthmatic subjects with a greater percentage of male patients (50.4% vs. Q 37.8% and V 39.8%, respectively), a higher number of asthma exacerbations (4.00 vs. Q 2.02 and V 2.01, respectively), a greater blood eosinophil count (400.0 cells/µL vs. Q 250 cells/µL and V 280 cells/µL, respectively), and especially a much higher percentage of patients presented with CRSwNP (61.4% vs. Q 22.9% and V 32.0%, respectively).

Dupilumab significantly reduced asthma symptoms, as demonstrated by the significant increase in ACT score, which after 24 weeks of biological therapy reached and exceeded the threshold of 20, which expresses a satisfactory control of asthma symptoms, despite the baseline pre-treatment score stood at a rather low value of 14. This result confirms, in a real-life context, the data reported by the Liberty Asthma QUEST trial, in which the ACQ (Asthma Control Questionnaire) was utilized. However, compared to the ACQ test, the ACT questionnaire administered by us seems to be more appropriate to respond to the needs of practicality and easiness of completion wished by patients who refer to our severe asthma assessment centers. In fact, ACT is clearly preferred to ACQ in the few real-life studies that have recently evaluated the efficacy of dupilumab in the biological therapy of severe asthma (15–19).

In patients with both asthma and nasal polyposis, dupilumab elicited a significant improvement in the score of SNOT-22 questionnaire. In such a group of subjects, this result was associated with a complete prevention of the relapses of nasal polyposis. These findings corroborate in a real-life setting the efficacy of dupilumab in the treatment of CRSwNP, previously demonstrated by the Liberty NP trials SINUS-24 and SINUS-52 (14).

In addition to the clinical effects, the results of our study regarding the respiratory function are also of marked relevance. Indeed, after 6 months of therapy dupilumab significantly increased FEV_1_, and also incremented FVC and FEF_25-75_. These findings indicate that dupilumab can improve lung function by increasing airway patency from the central proximal sector to the distal periphery of the respiratory tree. The clinical and functional results of the present observational study strongly suggest that clinical remission was achieved by a relevant number of our patients. Although this very important therapeutic target should be evaluated after 12 months of continuous treatment, already after 6 months we noticed that 47.24% of our patients reached the criteria of clinical remission, including significant improvements in asthma exacerbations, OCS intake, symptom control and lung function (32, 33). Such a real-life observation further confirms that dupilumab is characterized by a very fast onset of its therapeutic action, as already shown by previous data referring to the short-term clinical, functional and biological effects of this monoclonal antibody (18, 36). However, a few weeks of observation do not allow to assess the effects of dupilumab on severe asthma exacerbations. Thus, we decided to prolong up to 6 months the last time point for evaluation of dupilumab efficacy. Indeed, this approach made it possible to appreciate the impressive reduction of asthma exacerbations induced by dupilumab.

With regard to the biomarkers of type 2 inflammation, dupilumab dramatically reduced the concentration of FeNO. This outcome is closely linked to the ability of dupilumab to antagonize at the receptor level the biological activities of IL-4 and IL-13, the latter being responsible for the induction of iNOS expression in the bronchial epithelium (37, 38). Indeed, it is reasonable that inhibition of iNOS-dependent FeNO production, induced by dupilumab in airway epithelial cells, leads to relevant decrements of FeNO levels. FeNO is a reliable indicator of type 2 inflammation, and FeNO levels correlate with asthma severity, deterioration of respiratory function, and risk of asthma exacerbations (39). Furthermore, FeNO represents a valuable aid in guiding the choice and monitoring of biological treatments for severe asthma, within the context of a personalized therapeutic approach, based on the treatable traits pertinent to specific inflammatory pheno-endotypes (40).

Hence, the efficacy of dupilumab in inhibiting the pathobiologic mechanisms underlying type 2 inflammation, strongly dependent on IL-4 and IL-13 actions, explains the extension of the therapeutic effects of this monoclonal antibody to both asthma and nasal polyposis in our patients. In fact, asthma and CRSwNP share common cellular and molecular pathogenic substrates (41), which outline a very good responsiveness to dupilumab. In our observational investigation, the add-on biological therapy with dupilumab provided similar patterns of efficacy in allergic and non-allergic patients, suffering from severe asthma and possibly also from nasal polyposis. Indeed, dupilumab induced overlapping clinical and functional effects in subjects characterized by positive or negative skin prick tests. This is probably due to the specific properties of the mechanism of action of dupilumab, which by blocking IL-4 and IL-13 receptors effectively intercepts the pathogenic pathways responsible for type 2 inflammation sustained by either allergic or non-allergic traits. In particular, by neutralizing the biological activities of IL-4 and IL-13, dupilumab inhibits the functions of the main cells producing these cytokines, including Th2 lymphocytes and type 2 innate lymphoid cells (ILC2) (42). In this way dupilumab interrupts the close interactions between innate immunity and acquired adaptive immunity, mediated by the intercellular crosstalk between ILC2 and Th2 cells, which underlies the development and progression of type 2 inflammation characterizing many cases of severe asthma and nasal polyposis, driven by either allergic or non-allergic events (41, 43, 44).

Another interesting aspect of our real-life study concerns the finding of a greater decrease in FeNO values ​​detected in allergic patients, compared to non-allergic ones. This result suggests that patients characterized by a higher expression of multiple endotypic traits referable to type 2 inflammation, respond to dupilumab treatment by experiencing a greater decrement in FeNO levels. On the other hand, IL-4 and IL-13 are intensely involved in the cellular pathophysiology of type 2 inflammation, which could imply an enhanced predisposition of allergic patients to dupilumab-induced FeNO reduction. Furthermore, we did not detect significant correlations between serum IgE levels and the clinical and functional effects of dupilumab, whose therapeutic activity does not appear to be affected by the presence or absence of an atopic state. In our case series, the clinical and functional effects of dupilumab also occurred without substantial differences among severe asthmatic patients who presented or did not manifest the comorbidity of nasal polyposis. However, compared to patients without nasal polyps, we observed a greater reduction in FeNO values ​​in subjects with severe asthma and concomitant nasal polyposis. This suggests that patients characterized by type 2 inflammation involving both upper and lower airways are more susceptible to the therapeutic action of dupilumab. Therefore, it is plausible to speculate that the coexistence of severe asthma and nasal polyposis could be associated with a higher expression of IL-4 and IL-13 in the airways of patients reporting both these diseases, who would therefore respond to dupilumab with a more relevant decrease in FeNO levels. Finally, our real-life evaluation shows an excellent tolerability and safety profile of dupilumab, which did not induce significant adverse events. Differently from what occurred in some individuals recruited in the Liberty Asthma QUEST and Liberty Asthma VENTURE trials, though not confirmed by the open label extension TRAVERSE study, no increases in blood levels of eosinophils were found in our patients. Based on the specific mechanism of action of dupilumab, it is thus possible to explain the lack of effects of this drug on the number of blood eosinophils, as we report. In fact, dupilumab acts as a highly efficient dual receptor antagonist of IL-4 and IL-13, but does not interfere with the biological activity of IL-5, which is the main cytokine responsible for the maturation, activation, proliferation and survival of eosinophils (45).

In conclusion, the present observational study confirms and expands, in the real-life of pulmonary clinical practice, the data reported by randomized controlled trials investigating the efficacy of dupilumab in the treatment of severe asthma. In particular, we herein show that after 6 months of treatment this biological therapy had a very positive impact on asthma exacerbations, OCS consumption, symptom control in both asthma and nasal polyposis, respiratory function and FeNO levels. Such findings have been recently extended up to one year by the results of other real-world clinical investigations (46–48). In addition, we also detected these therapeutic benefits in both allergic and non-allergic patients, as well as in asthmatics with or without nasal polyposis. Therefore, our results further consolidate the strategic position of dupilumab in its role as an excellent therapeutic option currently available within the context of modern biological treatments of severe asthma and CRSwNP, frequently driven by type 2 airway inflammation.

## Data availability statement

The raw data supporting the conclusions of this article will be made available by the authors, without undue reservation.

## Ethics statement

The studies involving human participants were reviewed and approved by Local Ethics Committee of Calabria Region, Italy (Catanzaro, Italy; document n. 182 – 20 May 2021). The patients/participants provided their written informed consent to participate in this study.

## Author contributions

All authors contributed to data analysis, drafting or revising the article, have agreed on the journal to which the article will be submitted, gave final approval of the version to be published, and agree to be accountable for all aspects of the work.
